# Feasibility and Limitations of Vaccine Two-Dimensional Barcoding Using Mobile Devices

**DOI:** 10.2196/jmir.5591

**Published:** 2016-06-23

**Authors:** Cameron Bell, Julien Guerinet, Katherine M Atkinson, Kumanan Wilson

**Affiliations:** ^1^ Ottawa Hospital Research Institute Clinical Epidemiology Program Ottawa, ON Canada; ^2^ Karolinska Institutet Department of Public Health Sciences Stockholm Sweden; ^3^ University of Ottawa Departments of Medicine, Epidemiology and Community Medicine Ottawa, ON Canada

**Keywords:** vaccines, feasibility studies, immunization, automatic data processing, cell phones, vaccinations/standards

## Abstract

**Background:**

Two-dimensional (2D) barcoding has the potential to enhance documentation of vaccine encounters at the point of care. However, this is currently limited to environments equipped with dedicated barcode scanners and compatible record systems. Mobile devices may present a cost-effective alternative to leverage 2D vaccine vial barcodes and improve vaccine product-specific information residing in digital health records.

**Objective:**

Mobile devices have the potential to capture product-specific information from 2D vaccine vial barcodes. We sought to examine the feasibility, performance, and potential limitations of scanning 2D barcodes on vaccine vials using 4 different mobile phones.

**Methods:**

A unique barcode scanning app was developed for Android and iOS operating systems. The impact of 4 variables on the scan success rate, data accuracy, and time to scan were examined: barcode size, curvature, fading, and ambient lighting conditions. Two experimenters performed 4 trials 10 times each, amounting to a total of 2160 barcode scan attempts.

**Results:**

Of the 1832 successful scans performed in this evaluation, zero produced incorrect data. Five-millimeter barcodes were the slowest to scan, although only by 0.5 seconds on average. Barcodes with up to 50% fading had a 100% success rate, but success rate deteriorated beyond 60% fading. Curved barcodes took longer to scan compared with flat, but success rate deterioration was only observed at a vial diameter of 10 mm. Light conditions did not affect success rate or scan time between 500 lux and 20 lux. Conditions below 20 lux impeded the device’s ability to scan successfully. Variability in scan time was observed across devices in all trials performed.

**Conclusions:**

2D vaccine barcoding is possible using mobile devices and is successful under the majority of conditions examined. Manufacturers utilizing 2D barcodes should take into consideration the impact of factors that limit scan success rates. Future studies should evaluate the effect of mobile barcoding on workflow and vaccine administrator acceptance.

## Introduction

As digital health infrastructure evolves, the inclusion of product-specific identifiers in electronic health records will become of greater importance. This is particularly relevant for immunization practice where lot numbers in patient records are essential for the evaluation and surveillance of vaccine safety and effectiveness at the product level. However, product-specific identifiers are often recorded by hand, resulting in missing or inaccurate information. Missing data are known to produce gaps in communication between health care providers, increasing the potential for poor care coordination and medical errors [1]. Examination of children’s immunization records reveals transcription errors (in some cases more than 10%), administration of look-alike or sound-alike products, sibling confusion, and repeat immunization [2].

Vaccine products that protect against the same diseases are not necessarily the same formulation. The differentiation between vaccine products in vaccination records is essential for evaluations of the safety and effectiveness of vaccines. In order to identify different vaccine products, two-dimensional (2D) barcodes are often printed on vaccine vials. The most commonly employed 2D barcode standard for vaccine vials is the DataMatrix. At 2-3 mm^2^, DataMatrix barcodes can store up to 50 alphanumeric characters, making them capable of containing a Global Trade Item Number (GTIN), an expiration date, and a lot number, in an image small enough to be printed directly on unit-of-use product labels [3]. GTINs are identification numbers that are used to identify products all over the world. 2D barcode scanning has the potential to play an important role in automating the identification of vaccines such that they can be included in electronic health records efficiently and with fewer errors [4].

Barcode scanning of vaccine products is not widely implemented, although preliminary implementation pilots are positive, showing improvements in data completeness and reduction in data errors [5]. A time-motion study demonstrated that scanning 2D barcoded vaccines could reduce immunization documentation time by 36-39 seconds per dose [6]. Training requirements and process flow issues, access to and adoption of technology, and resistance to change are known barriers to the implementation of barcode scanning within health facilities [7].

Barcode scanning facilitated by mobile devices such as mobile phones could potentially increase the amount of vaccine product-specific information residing in digital health records by making barcode scanning more readily accessible to both health care providers and patients. Health care providers could use the mobile device they already own as a scanner instead of purchasing a handheld scanner. Additionally, health care providers working in remote areas where carrying a handheld scanner is not feasible would likely still be able to use their mobile device to capture data. Enabling patients to capture their own product-specific records could also be beneficial, especially within immunization where parents are often responsible for maintaining their children’s immunization data. Although it is unlikely that a parent would be given the vaccine vial to scan, a barcode could be provided to a patient on a vaccine information sheet, which the patient could scan to capture the information into a personal vaccination record app. The feasibility of mobile barcode scanning of vaccine vials and its limitations remain uncertain. Our objective in this study was to examine the feasibility, potential limitations, and variability in performance of scanning vaccine vial barcodes using mobile phones.

## Methods

### Objectives

We sought to determine whether mobile phones are capable of accurately scanning 2D vaccine barcodes. We specifically examined the impact of barcode size, curvature, fading, and lighting on the ability to successfully scan 2D barcodes, as well as how barcode scanning ability varies among different mobile devices.

### Study Setting and Variables Examined

A mobile phone app was developed for iOS and Android platforms that scans barcodes and records whether the scan was successful within an allotted amount of time. The app was developed by programmers at the Ottawa Hospital Research Institute specifically to perform this study. The time to scan the barcode was also recorded. The app was loaded onto 4 different mobile phone devices that were state of the art in mid to late 2013: the iPhone 5, the Samsung Galaxy S4, the Nexus 5, and the Nexus 7 (Table 1) [8-11]. The mobile app developed was used to perform a validation study on the ability of the devices to scan the 2D barcodes under a variety of laboratory conditions.

The experiment was divided into 4 trials, each evaluating the effect of 1 variable (barcode size, fading, curvature, and ambient lighting) on the scannability of perfectly printed 2D DataMatrix barcodes as recorded by the app (Table 1). A series of barcode samples was produced for each trial and printed on standard printer paper using an ink-jet printer (Multimedia Appendix 1).

**Table 1:** Trial conditions.

Trial	Measuring	Size	Curvature	Fading	Ambient light	No. of scans
1	Size	Varied in 0.5-mm increments between 5 mm and 9 mm	Flat	None	500 lux	9
2	Fading	7 mm	Flat	Varied in 10% increments between 0% and 90%	500 lux	10
3	Curvature	7 mm	0 mm (flat), 10 mm, 15 mm, 17 mm	None	500 lux	4
4	Ambient light	7 mm	Flat	None	5, 20, 150, 500 lux	4

The study was performed in Ottawa, Canada. All trials were performed in a room with no natural light, with a light source fixed at a specific illuminance. Illuminance is a measure of the quantity of light travelling past a surface and was measured using a lux meter adjacent to the location where the vials were scanned. Preliminary experiments were performed before the validation study in order to characterize the range of values for each independent variable. A baseline value was determined for each variable at the value where the variable no longer had an effect on the ability of all devices to scan barcodes one hundred percent of the time. Holding these baseline values constant for 3 of the variables allowed us to isolate the effect of the fourth variable. The baseline values were determined by repeatedly attempting to scan barcodes while increasing the variable parameter until 10 successive scan attempts succeeded, for each of the 4 devices.

During all of the trials, except that which evaluated the ambient lighting variable, the illuminance was fixed as close to 500 lux as possible. An illuminance of 500 lux was established as the baseline illuminance in our preliminary experiments. The Canadian Occupational Health and Safety Regulations recommend illuminance levels of 1000 lux in examination and treatment rooms and 500 lux in other health care environments [12].

#### Size

To evaluate the impact of size on barcode scannability, the sample set consisted of a series of 9 barcodes decreasing in size by 0.5 mm from 9 mm to 5 mm. The value of 5 mm was chosen as the lower limit, as this is the barcode size present on single-dose vaccine syringes. A value of 7 mm was identified as the baseline size used to eliminate the effect of size on the other trials.

#### Fading

For the fading variable, the sample set consisted of a series of 10 barcodes. Fading was applied such that a barcode with 0% fading would be printed with full black color and a barcode with 100% fading would be invisible. The series used for the trial consisted of barcodes with increasing fading in increments of 10% from 0% to 90%. The upper limit was 90% as at 100% the barcode is not visible.

#### Curvature

To evaluate curvature, 4 barcodes with a uniform size of 7 mm were printed on adhesive paper and pressed onto vaccine vials with diameters of 10 mm, 15 mm, and 17 mm. These diameters correspond to those of the Sanofi Pasteur 0.5-mL-dose syringe, 1-mL-dose vial, and 0.5-mL-dose vial, respectively. A fourth barcode was printed on a flat surface. All other trials were performed entirely with flat barcodes.

#### Lighting

To evaluate ambient lighting, ideal 7-mm printed barcodes were scanned at 4 illuminance levels. A lux meter was used to measure the illuminance in the immediate area where the barcode was scanned. A dimmer was used to adjust the lighting in the room to specific light intensities, as may be experienced in different clinical settings. The 4 illuminance levels tested were 500, 150, 20, and 0 lux. The illuminance 20 lux was chosen because in our initial experiments scanning became difficult around this point. Lastly, we chose 5 lux to simulate a near pitch-dark environment.

### Study Procedure

The first page of the app allows the experimenters to select the trial they want to perform and to input their name (Multimedia Appendix 2, Screenshot 1). For all of the trials except the ambient lighting trial, the experimenter is required to enter the illuminance measurement at which the trial is being performed.

To complete a single trial, an experimenter scanned each barcode in the series once. Each trial was performed 10 times on each of the 4 devices, by 2 independent experimenters. The experimenters went through a training period where they each performed each trial twice to familiarize themselves with the scanning procedure. For all trials except the curvature trial, the paper containing the barcode was fastened to a flat surface. For the curvature trial, the vials to which the barcodes were adhered were fastened to the surface with the barcodes facing up.

When the experimenter begins a trial, a screen is shown that indicates how many scans are remaining in the trial and which barcode must be scanned next (Multimedia Appendix 2, Screenshot 2). When the user taps the scan button, the device must be in the user’s hand, which must be resting on the table surface 8 inches to the right of the sample. Only after the scan button has been pressed may the user move the device to attempt to scan the barcode. This was done to eliminate disparities between scans due to the experimenter maintaining the exact position and height of the device, which promotes subsequent scans being far quicker than the initial scan.

### Analysis

A scan is defined as successful when the scanner reads the correct code printed in the barcode within 10 seconds. We chose the limit of 10 seconds as we expect this to be the maximum amount of time a user would continue attempting to scan a vial without success [13]. In addition to the 4 variables mentioned above, we also looked at the percentage of scans that succeeded but returned an incorrect value. To measure this, the app checks whether each scan returns the value encoded in the barcodes in the sample and records this as part of the scan data (Table 2 in Multimedia Appendix 3). The app outputs the results of each trial as a comma-separated vector file. The interrater reliability was calculated between the 2 raters using the two-way random, single measure, intraclass correlation (ICC). The values for each subject compared in the ICC calculation are the scan success rates (out of 10) of each rater for each subject. A subject, for our purpose, is defined as the set of 10 scans that have the same variables: trial type, sequence number, and device.

## Results

### Accuracy of Barcode Scanning

Each experimenter performed all 4 trials 10 times each, amounting to a total number of 2160 barcode scan attempts. Out of the 1832 successful scans there were zero scans that registered as successful but produced incorrect data (Table 2).

**Table 2:** Total successful scans for all trial conditions and by device.

Trial condition		Total success rate, %		Successful scans by device, n							
				Nexus 7		iPhone 5		Nexus 5		Samsung Galaxy S4	
Size, mm											
	5		100.00		20		20		20		20
	5.5		100.00		20		20		20		20
	6		100.00		20		20		20		20
	6.5		100.00		20		20		20		20
	7		100.00		20		20		20		20
	7.5		100.00		20		20		20		20
	8		100.00		20		20		20		20
	8.5		100.00		20		20		20		20
	9		100.00		20		20		20		20
Fading, %											
	0		100.00		20		20		20		20
	10		100.00		20		20		20		20
	20		100.00		20		20		20		20
	30		100.00		20		20		20		20
	40		100.00		20		20		20		20
	50		100.00		20		20		20		20
	60		77.50		14		16		13		19
	70		1.25		0		0		0		1
	80		0.00		0		0		0		0
	90		0.00		0		0		0		0
Curvature, mm											
	0		100.00		20		20		20		20
	10		88.75		20		18		17		16
	15		100.00		20		20		20		20
	17		98.75		20		20		20		19
Illuminance, lux											
	500		100.00		20		20		20		20
	150		100.00		20		20		20		20
	20		95.00		20		20		18		18
	5		28.75		0		0		7		16

### Interrater Reliability

The ICC between the 2 raters was observed to be .947 with a confidence interval of .921 to .964. Table 3 presents the number of successful scans per rater by device for each subject. The most significant deviation between the 2 raters occurs during the fading trial at 60% fading, where rater 1 is more successful on every device than rater 2 by approximately 180% on average. The second largest deviations occur in the curvature and lighting trials on the Samsung Galaxy S4 device, where in both cases rater 1 succeeds on every attempt and rater 2 succeeds on 86.25% of the attempts. For each trial, the point at which interrater reliability begins to deteriorate likely borders on the limitations of the technology for practical use.

**Table 3:** Total number of successful scans per device and rater.

Trial Condition		Successful Scans by Device and Rater							
		Nexus 7		iPhone 5		Nexus 5		Samsung Galaxy S4	
		Rater 1	Rater 2	Rater 1	Rater 2	Rater 1	Rater 2	Rater 1	Rater 2
Size, mm									
	5	10	10	10	10	10	10	10	10
	5.5	10	10	10	10	10	10	10	10
	6	10	10	10	10	10	10	10	10
	6.5	10	10	10	10	10	10	10	10
	7	10	10	10	10	10	10	10	10
	7.5	10	10	10	10	10	10	10	10
	8	10	10	10	10	10	10	10	10
	8.5	10	10	10	10	10	10	10	10
	9	10	10	10	10	10	10	10	10
Fading, %									
	0	10	10	10	10	10	10	10	10
	0.1	10	10	10	10	10	10	10	10
	0.2	10	10	10	10	10	10	10	10
	0.3	10	10	10	10	10	10	10	10
	0.4	10	10	10	10	10	10	10	10
	0.5	10	10	10	10	10	10	10	10
	0.6	10	4	10	6	9	4	10	9
	0.7	0	0	0	0	0	0	1	0
	0.8	0	0	0	0	0	0	0	0
	0.9	0	0	0	0	0	0	0	0
Curvature, mm									
	0	10	10	10	10	10	10	10	10
	10	10	10	10	8	8	9	10	6
	15	10	10	10	10	10	10	10	10
	17	10	10	10	10	10	10	10	9
Illuminance, lux									
	5	0	0	0	0	3	4	10	6
	20	10	10	10	10	8	10	10	8
	150	10	10	10	10	10	10	10	10
	500	10	10	10	10	10	10	10	10

### Impact of Size

**Figure 1:**
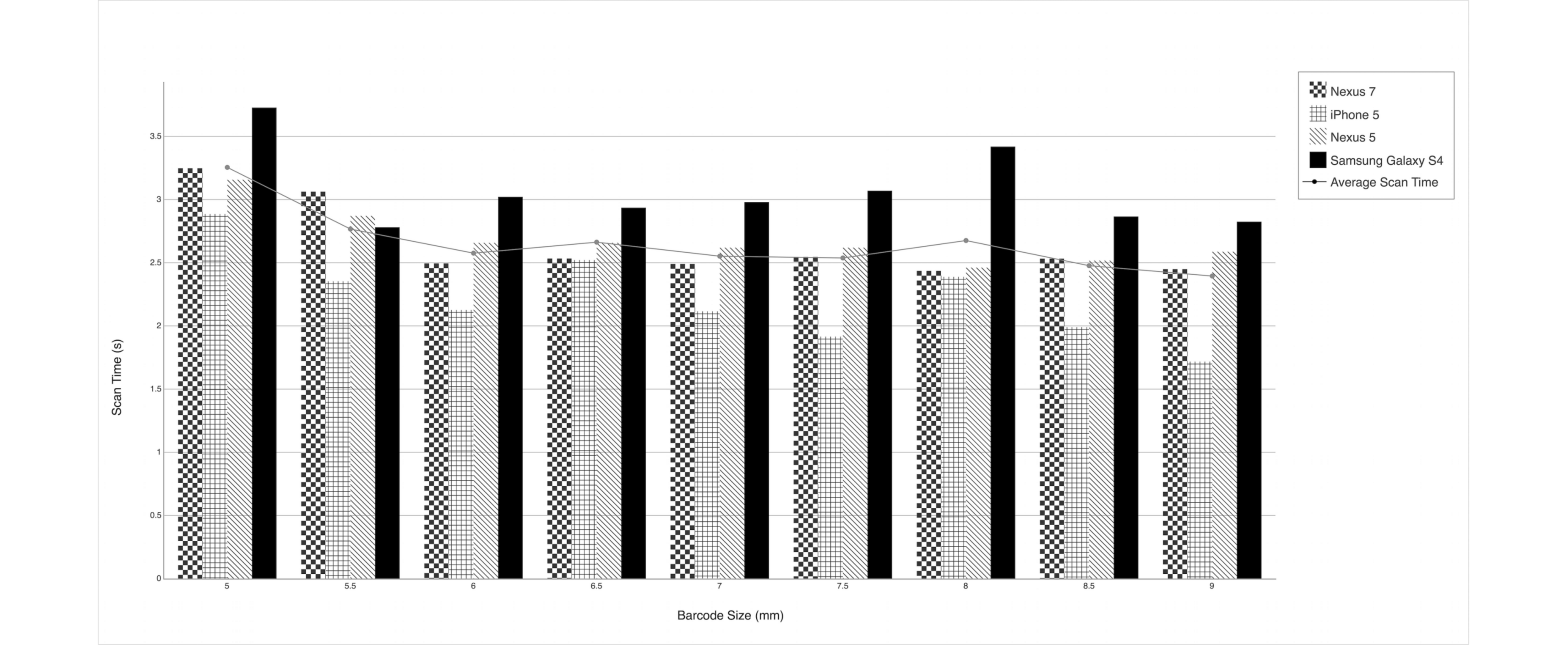
Scan Time by Barcode Size and Device.

The size trial yielded a 100% scan success rate, meaning that every scan attempt succeeded before timing out (Table 2). The results of the size trial indicated that 5-mm barcodes took longer to scan than any of the other barcode sizes we tested, although only by 0.5 seconds on average. Figure 1 shows the average scan time across all devices and experimenters for each barcode size tested.

When we examined how scan time differed among devices (Figure 1), the Samsung Galaxy S4 tended to have consistently longer scan times (0.55 seconds more) than the other devices. The iPhone consistently had the lowest average scan time.

### Impact of Fading

The results of the fading trial demonstrated that fading begins to affect scan time and overall scannability at 60% and becomes nearly impossible at 70%. Table 2 shows that up until 50% there is a 100% scan success rate. At 60% fading, however, the scan success rate decreases to ~78%.

**Figure 2:**
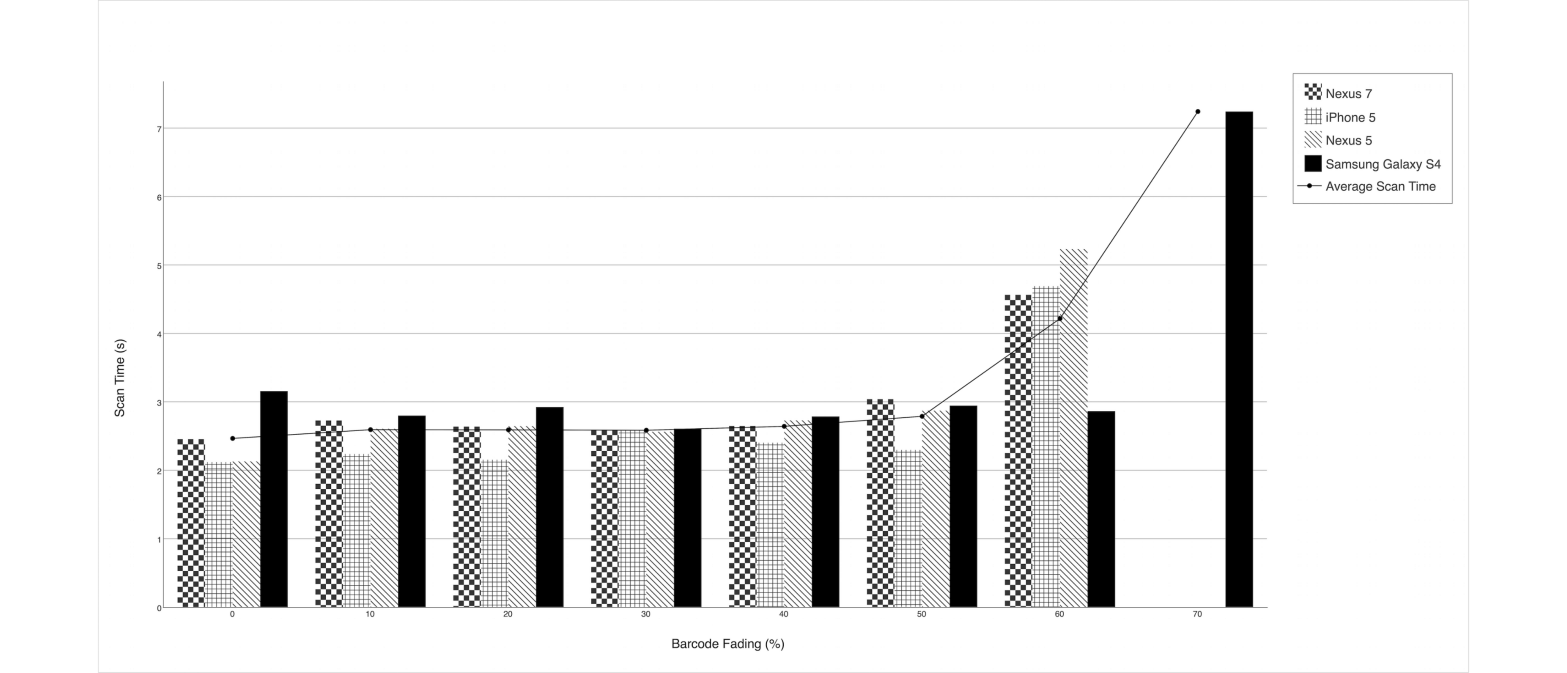
Scan Time by Barcode Fading and Device.

The Samsung Galaxy S4 device exhibited superior scan time and scan success percentage and was the only device to scan the 70% faded barcode (Figure 2,Table 2). Up until the 60% faded barcode, the performance of all the other devices was mostly uniform.

### Impact of Curvature

**Figure 3:**
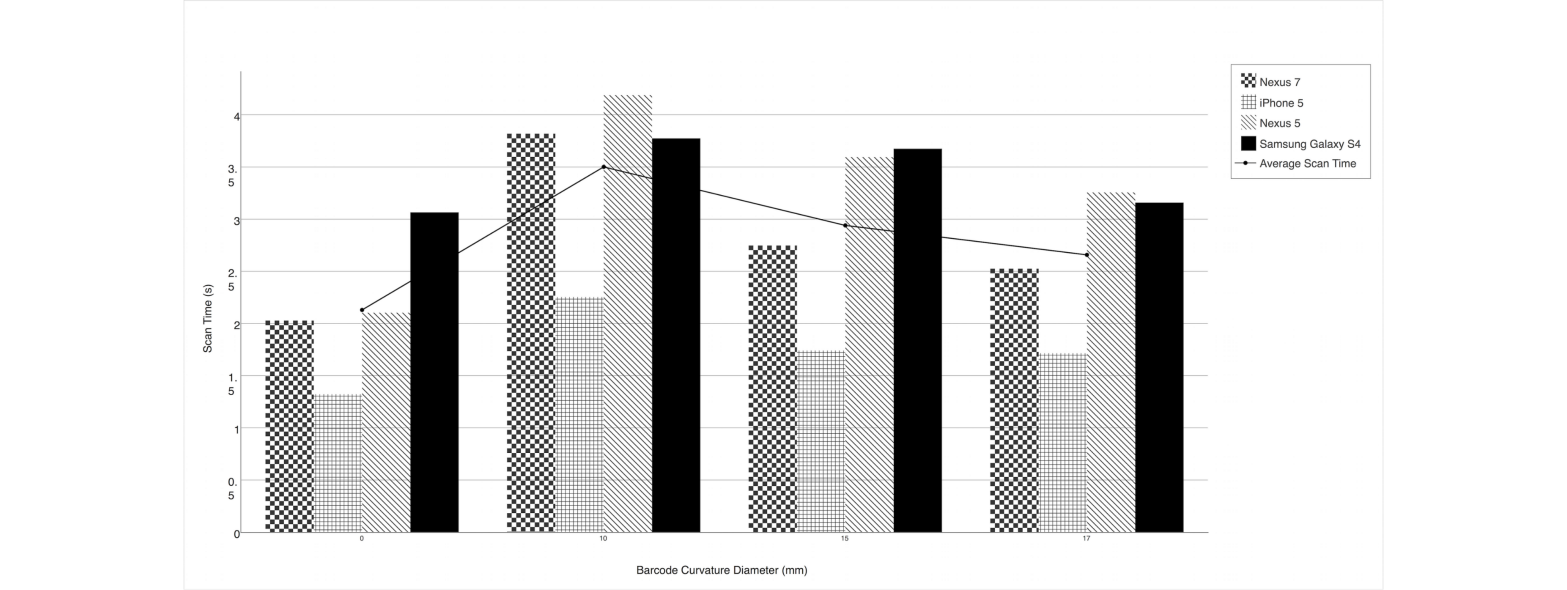
Scan Time by Barcode Curvature and Device.

The results of the curvature trial can be seen in Figure 3. The curvature with the smallest diameter (10 mm) saw the longest scan time and lowest scan success percentage at 3.5 seconds and 88%, respectively (Table 2). On average, all of the curved barcodes took longer to scan than flat barcodes; however, the scan success rate remained at approximately 100% with the exception of the barcode with the 10-mm diameter of curvature. When examining scan time by device presented in Figure 3, the iPhone handled curvature better than any of the other devices with an average scan time approximately 1 full second less than the average. The Samsung Galaxy S4 had the longest scan time, with an average scan time 0.6 seconds higher than the average.

### Impact of Lighting

**Figure 4:**
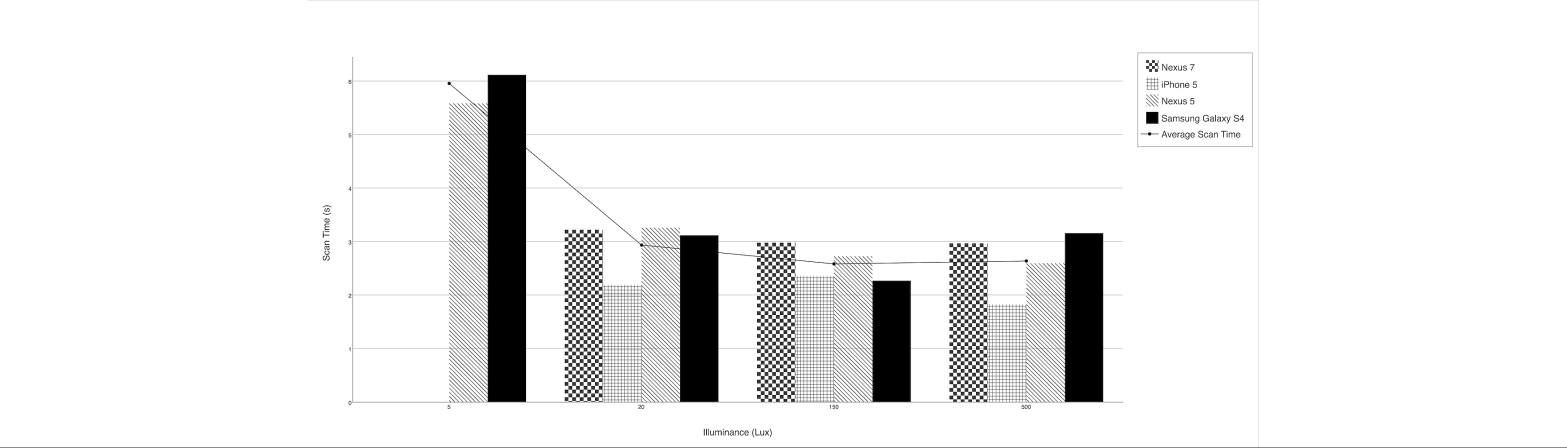
Scan time by barcode illuminance and device.

Figure 4 shows the effect of varying the illuminance at the barcode on scan time. Scannability only degraded significantly once illuminance dropped below 20 lux (Table 2). The Samsung Galaxy S4 and Nexus 5 devices performed the best at the lowest lighting condition, whereas the iPhone and Nexus 7 were completely unable to scan the barcode at 5 lux. For the top 3 illuminance levels, the performance of the devices was mostly uniform with the iPhone exhibiting slightly shorter scan times, as it tended to do throughout the experiment (Figure 4).

## Discussion

We successfully developed a mechanism for scanning 2D barcodes using mobile devices. We had a 100% data accuracy rate for all barcodes successfully scanned. When examining factors potentially limiting 2D barcode scanning, our study found that, given ideally printed barcodes and using modern mobile phones, the following are true:

1. Barcodes as small as 5 mm can be scanned reliably. The average scan time could be marginally increased by using barcodes larger than 5 mm.

2. Scannability begins to decrease significantly when the barcode has faded past 50%.

3. Curvature begins to affect scannability between 10- and 15-mm diameters.

4. Illuminance begins to deplete scannability around 20 lux.

Our results suggest that modern mobile phones should be able to scan barcodes printed on vaccine vials and other packaging, assuming those barcodes are printed without errors, larger than or equal to 5 mm, and do not exhibit fading greater than 50%.

Performance was mostly uniform across all devices tested. It became evident that scan time was mostly dependent on the properties of the software program as opposed to the hardware properties of the device. For instance, the iPhone outperformed any other device on almost every trial when looking at scan time; however, this is most likely due to the settings of the auto-focus timer that is responsible for periodically adjusting the focus of the camera in order to refocus on the subject. The auto-focus timer on the Android devices was approximately 2 seconds, whereas the auto-focus timer on the iPhone was approximately 1.5 seconds. It is this disparity that is largely responsible for the faster scan times of the iPhone. Despite device variation, our average scan time results were similar to what was found by Pereira et al [14] who utilized scanners retailing for almost US $800 (PowerScan D8530 Handheld Scanner, Datalogic Mobile Inc).

Although it was to be expected, the absolute absence of data errors (successful scans yielding incorrect data) is an important outcome as it confirms the reliability of mobile barcode scanning with respect to data integrity. Data errors are possible, especially in a live setting where barcodes are not necessarily printed with perfect precision. In most jurisdictions, parents are still required to maintain paper records of their children’s immunizations and some surveys indicate that more than a quarter of these records are incomplete, contain data errors, or are lost completely [15]. Accurate tracking of an individual’s immunization history is important in order to prevent duplicate immunizations and when proof of immunization is required for school, day care, and so on. Recording errors are common due to vaccine or patient-related human factors [16]. As individuals increasingly receive vaccines from multiple providers, it is important that individuals maintain accurate records as they will often be viewed as the single source of truth when it comes to their immunization status [17]. Research demonstrates that increasing responsibility and control over one’s own health records, including immunization history, fosters greater engagement within the health care system and increases knowledge about personal health [18]. As registries in many jurisdictions underestimate coverage [19,20], any mechanism that empowers the individual to record and report immunization encounters has the potential to improve immunization programs.

Although this study focusses on vaccine vials, 2D DataMatrix barcodes are being used increasingly to identify other medications and medical devices. One application that could be of particular interest to the public would be the use of barcode scanning to include product expiry information in consumer mobile apps. A person who depends on an inhaler could use his or her mobile device to scan the 2D barcode and capture the expiry date into an app that will remind the user to renew the prescription before it expires.

Mobile barcode scanning, like other mobile technologies [21], could provide value to practitioners. This may include data entry efficiencies for product-specific information at the point of care, resulting in fewer information gaps [22]. When barcode scanning is not available, physicians and nurses must read the information off of the vaccine packaging or vial and enter the information by either manually typing or selecting choices from drop-down menus. Both of these methods have been shown to produce more errors in hospital electronic medical records compared with barcoding methods [23].

To the best of our knowledge there have been no other studies that evaluate the limits of mobile barcode scanning. This study benefits from its use of 2 experimenters. We observed high interrater reliability between the 2 experimenters, which suggests that our results are reproducible.

A limitation of this study is that we did not evaluate the ability of mobile devices to scan the barcodes on real vials. There are three potential problems: first, from observation we know that the barcodes on some vaccine vials are not printed with sufficient quality to permit scanning with mobile devices. Some barcodes are printed with defects and many have been shown to exhibit fading of the print [24]. It is likely that the print quality will have to improve before scanning with mobile devices is entirely feasible. Second, our study evaluates the 4 variables independently, whereas when scanning an actual vial there will be an interaction of variables. For instance, it is possible that in low-light environments, the threshold, at which a faded barcode becomes impossible to scan, is higher. Consequently, the study benefits from observing each variable independently in that we can say definitively what impact each variable has on scannability and our results are not skewed by the imperfect print quality of the barcodes on actual vials. In addition, to thoroughly evaluate the interdependence of each variable would require hundreds of thousands of scans, which may not be feasible. Last, the surface on which the barcode is printed and the reflectiveness property of the material would likely have an impact on scannability as well and would need to be evaluated as a fifth variable.

Another limitation of this study was the scope of devices we tested. The devices we used were state of the art in mid-2013. There exists a wide range of devices of lower quality both in computing power and in camera quality. Including some of these lower-quality devices in the study may have given a better indication of the lower limit of scannability. Since 2013, mobile devices have improved considerably, allowing for a shift away from relying on desktop processors [25]. We have made the reasonable assumption that newer phones that have higher specifications than the devices we tested will have equivalent or improved scanning performance.

Immunization information systems and registries are critical to the success of immunization programs [26] and become more powerful when they include product-specific identifiers. Mobile barcode scanning could serve as a mechanism to increase the amount of product-specific information captured in these systems by lowering the barriers to entry of barcode scanning at point of care [17]. We propose that there are two further components to the evaluation of mobile barcode scanning as an approach to the automated identification of vaccine products. First, an evaluation of mobile barcode scanning performance using real vaccine vials will be important to determine whether barcodes on vaccine products are being printed with sufficient precision to facilitate barcode scanning. Second, a usability study is necessary to determine whether mobile barcode scanning by individuals with little training is feasible and advantageous to manual text entry or drop-down selections. Although these two evaluations could tell us that mobile devices could in fact be used to facilitate barcode scanning, the greatest obstacle to the use of this technology will be incentivizing individuals and practitioners to use it. A third evaluation should be performed to determine whether mobile barcode scanning can be integrated into the physician’s or nurse’s workflow when vaccinating a patient.

### Conclusions

This study has demonstrated that accurate 2D vaccine barcode scanning by mobile devices is possible and can be successful under the majority of laboratory conditions we examined. Within the context of vaccine barcoding in Canada, our results suggest that modern mobile phones should be able to scan barcodes printed on vaccine vials and packaging, assuming those barcodes are printed without errors, larger than or equal to 5 mm, and do not exhibit fading greater than 50%. Barcode scanning has been demonstrated to have a positive effect on the quality of health records [23], and mobile barcode scanning makes the technology available to a far greater number of health care providers and individuals than would otherwise have access to handheld scanners. The need for product- and lot-specific information to be captured when building immunization information systems suggests a need for increased access to barcode scanning, which this study suggests could be fulfilled using mobile devices. Mobile barcode scanning should be considered as an adjunct to barcode scanning using dedicated handheld scanners, and manufacturers utilizing barcodes should take into consideration the factors that limit scanning.

## Appendix

Multimedia Appendix 1 Barcode samples.

Multimedia Appendix 2 App Screenshots.

Multimedia Appendix 3 Device specifications and data captured per scan.

## Abbreviations

2D: two-dimensional
GTIN: Global Trade Item Number
ICC: intraclass correlation
