# Supplementary material for: Feasibility and Limitations of Vaccine Two-Dimensional Barcoding Using Mobile Devices
Source: J Med Internet Res. 2016 Jun 23;18(6):e143. doi: 10.2196/jmir.5591 (PMC4937181; doi:10.2196/jmir.5591)
Supplement: Multimedia Appendix 1 [file jmir_v18i6e143_app1.pdf]

MULTIMEDIA APPENDIX 2 – BARCODE SAMPLES

A full resolution version of this barcode sample can be accessed here: http://bit.ly/1Hz8kw6

5mm 0%	5.5mm 0%	6mm 0%	6.5mm 0%	7mm 0%	7.5mm 0%	8mm 0%	8.5mm 0%	9mm 0%
5mm 10%	5.5mm 10%	6mm 10%	6.5mm 10%	7mm 10%	7.5mm 10%	8mm 10%	8.5mm 10%	9mm 10%
5mm 20%	5.5mm 20%	6mm 20%	6.5mm 20%	7mm 20%	7.5mm 20%	8mm 20%	8.5mm 20%	9mm 20%
5mm 30%	5.5mm 30%	6mm 30%	6.5mm 30%	7mm 30%	7.5mm 30%	8mm 30%	8.5mm 30%	9mm 30%
5mm 40%	5.5mm 40%	6mm 40%	6.5mm 40%	7mm 40%	7.5mm 40%	8mm 40%	8.5mm 40%	9mm 40%
5mm 50%	5.5mm 50%	6mm 50%	6.5mm 50%	7mm 50%	7.5mm 50%	8mm 50%	8.5mm 50%	9mm 50%
5mm 60%	5.5mm 60%	6mm 60%	6.5mm 60%	7mm 60%	7.5mm 60%	8mm 60%	8.5mm 60%	9mm 60%
5mm 70%	5.5mm 70%	6mm 70%	6.5mm 70%	7mm 70%	7.5mm 70%	8mm 70%	8.5mm 70%	9mm 70%
5mm 80%	5.5mm 80%	6mm 80%	6.5mm 80%	7mm 80%	7.5mm 80%	8mm 80%	8.5mm 80%	9mm 80%
5mm 90%	5.5mm 90%	6mm 90%	6.5mm 90%	7mm 90%	7.5mm 90%	8mm 90%	8.5mm 90%	9mm 90%
