# Supplementary material for: Feasibility and Limitations of Vaccine Two-Dimensional Barcoding Using Mobile Devices
Source: J Med Internet Res. 2016 Jun 23;18(6):e143. doi: 10.2196/jmir.5591 (PMC4937181; doi:10.2196/jmir.5591)

Multimedia Appendix 3 – App screenshots

Screenshot 1 – Select trial screen in the developed barcode scanning experiment app.

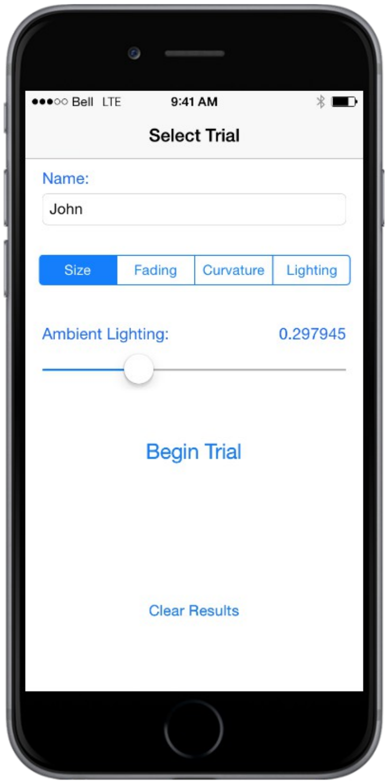

Screenshot 2 - Trial prompt screen in developed barcode scanning experiment app.

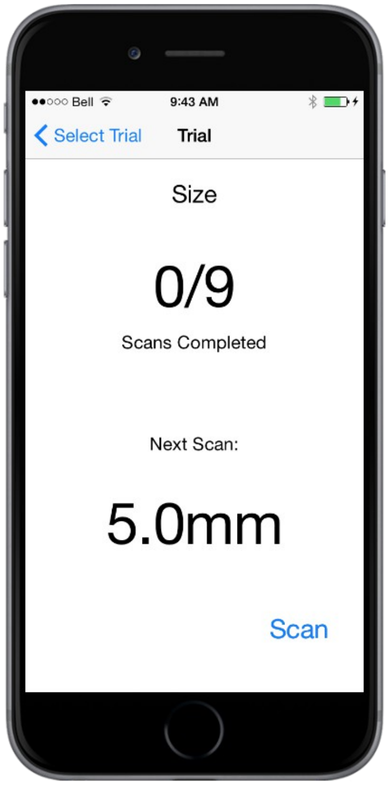

Supplement: Multimedia Appendix 2 [file jmir_v18i6e143_app2.pdf]
