# Supplementary material for: Feasibility and Limitations of Vaccine Two-Dimensional Barcoding Using Mobile Devices
Source: J Med Internet Res. 2016 Jun 23;18(6):e143. doi: 10.2196/jmir.5591 (PMC4937181; doi:10.2196/jmir.5591)
Supplement: Multimedia Appendix 3 [file jmir_v18i6e143_app3.pdf]

Multimedia Appendix 1 – Device specifications and data captured per scan

Table 1 – Test device specifications

Device Name	OS	OS version	Megapixel Count	Processor	Processor Speed
iPhone 5	iOS	8.2	8 MP, 3264 x 2448 pixels	Dual-core 1.3 GHz Swift (ARM v7-based)	1.3 GHz
Nexus 5	Android	5.1.0	8 MP, 3264 x 2448 pixels	Qualcomm MSM8974 Snapdragon 800 Quad-core 2.3 GHz Krait 400	2.3 GHz
Samsung Galaxy S4	Android	4.4.1	13 MP, 4128 x 3096 pixels	Qualcomm Snapdragon 600 APQ8064T	1.9 GHz
Nexus 7	Android	4.4.4	5 MP	Qualcomm Snapdragon S4 Pro APQ8064 1500 MHz	1.5 GHz

Table 2 – Example of data captured by application at each scan

Trial ID	Trial Type	Sequence #	Scan Name	Scan Success	Data Success	Scan Time	Lighting	Scan Date	Experimenter	Device
1434558810	0	0	5.0mm	1	1	3.486176	512	2015-06-17T12:33:30.936Z	John	iPhone5-1
